# Iconicity in the lab: a review of behavioral, developmental, and neuroimaging research into sound-symbolism

**DOI:** 10.3389/fpsyg.2015.01246

**Published:** 2015-08-24

**Authors:** Gwilym Lockwood, Mark Dingemanse

**Affiliations:** ^1^Neurobiology of Language Department, Max Planck Institute for Psycholinguistics, Nijmegen, Netherlands; ^2^Language and Cognition Department, Max Planck Institute for Psycholinguistics, Nijmegen, Netherlands

**Keywords:** iconicity, sound-symbolism, neuroimaging, psycholinguistics, linguistics, ideophones, synesthesia, cross-modal correspondence

## Abstract

This review covers experimental approaches to sound-symbolism—from infants to adults, and from Sapir’s foundational studies to twenty-first century product naming. It synthesizes recent behavioral, developmental, and neuroimaging work into a systematic overview of the cross-modal correspondences that underpin iconic links between form and meaning. It also identifies open questions and opportunities, showing how the future course of experimental iconicity research can benefit from an integrated interdisciplinary perspective. Combining insights from psychology and neuroscience with evidence from natural languages provides us with opportunities for the experimental investigation of the role of sound-symbolism in language learning, language processing, and communication. The review finishes by describing how hypothesis-testing and model-building will help contribute to a cumulative science of sound-symbolism in human language.

## Introduction

Despite the increasing acceptance and popularity of sound-symbolism research in recent years, many articles about sound-symbolism begin by defining it in opposition to arbitrariness. The traditional Saussurian (de Saussure, 1959) or Hockettian (Hockett, 1959) view of language is outlined, the strengths of arbitrariness as a productive and compositional system (Monaghan and Christiansen, 2006) are described, the psychological and neuroscientific models of language which are built around arbitrariness (Levelt et al., 1999; Friederici, 2002; Hickok and Poeppel, 2007; Hagoort, 2013) are enumerated… and with a flourish, the latest sound-symbolism research is uncovered to the reader. All is not what it seems!

This approach is certainly not without its uses; even relatively recently, the extent of sound-symbolism within any given language was dismissed as “vanishingly small” (Newmeyer, 1992), and so the prerogative of sound-symbolism researchers to point out the shortcomings and blind spots of an approach that sees language as strictly arbitrary is understandable. However, to continue to present sound-symbolism as an opponent to arbitrariness, rather than simply the opposite of arbitrariness, is unhelpful. The two systems are clearly happy enough to co-exist within language; with iconic links between sound/sign and meaning increasingly being accepted as a general property of language (Perniss et al., 2010; Perniss and Vigliocco, 2014), it is time for a more constructive perspective.

Despite the fast growing interest in iconicity in general (as witnessed for instance in studies of sign language and gesture), there is still a relative dearth of experimental research on sound-symbolism, especially when compared with the amount of psycholinguistic research based on arbitrary words. However, research into sound-symbolism has been steadfastly gaining influence in fields like linguistics, psycholinguistics and cognitive neuroscience, opening up new opportunities for theoretical and empirical progress. What is needed now is a perspective that unites these bodies of evidence and shows where they converge or diverge. This review article brings together experimental findings from a wide range of fields—from behavioral experiments to developmental work and neuroimaging studies—and shows that there is now an exciting opportunity to develop a holistic account of the communicative functions and causal mechanisms of sound-symbolism.

## Definitions and History

The discussion of arbitrariness *versus* sound-symbolism is nothing new. Plato’s *Cratylus* describes a debate between Cratylus and Hermogenes about the origin of names, with Cratylus arguing that names are meaningful in themselves and by nature, and Hermogenes arguing that names are merely signifiers^1^. Socrates, the umpire of the debate, acknowledges both points; he presents a Hamano-esque description of the “imitative significance of primary sounds corresponding to single letters of the alphabet” (Hamano, 1998), followed by the argument that any name, even if it is natural, cannot perfectly describe its referent and thus some degree of linguistic convention is inherent to all names (Sedley, 2003)^2^.

Arbitrariness and iconicity, “the source of more trouble than any other aspect of communicative behavior” (Hockett, 1959), continued to set themselves apart throughout the Middle Ages and well into the twentieth century. It was only in the middle of the twentieth century that arbitrariness was fully enshrined as the principle cornerstone of language, basing linguistic theory upon de Saussure’s (1959) posthumously translated and published work on the arbitrariness of the sign and Hockett’s (1959) assertion that arbitrariness is one of seven—later updated to 13 (Hockett, 1960)—key design features of human language.

### Competing Motivations for Arbitrariness and Sound-Symbolism

The strength of arbitrariness was identified as the ability to combine symbols into limitless conventional forms, giving language far more communicative power in terms of the range of concepts and relations it can express, while also explaining why different languages have different forms for the same concepts. Crucially though, Hockett (1960) also acknowledged that while the design feature arbitrariness gives limitless possibilities to communication, it also “has the disadvantage of being arbitrary.” This is a caveat with implications for learning and communication which has not always been addressed. Indeed, more recent studies have indicated that sound-symbolism and arbitrariness mutually pick up each other’s slack. Non-arbitrary form-to-meaning relationships facilitate learning as they are grounded in existing perceptual and cognitive systems (Cuskley and Kirby, 2013) and enable the grouping of similar words into categories (Farmer et al., 2006). Arbitrariness facilitates the learning of specific word meanings (Monaghan et al., 2011) and prevents the confusion of concepts which are similar but critically different (such as two almost identical mushrooms; one edible, one poisonous; Corballis, 2002).

A system based solely on arbitrariness would pose immense learning difficulties, with no link between linguistic form and human experience, and would make communication less direct and vivid; a system based solely on sound-symbolism would prevent specificity of communication because it can only offer limited conceptual distinctions (Bühler, 1990). The recognition that sound-symbolism and arbitrariness coexist in language is echoed in recent theoretical syntheses of arbitrariness and iconicity (Perniss et al., 2010; Perniss and Vigliocco, 2014). They can coexist because each brings its own advantages for learning words and using them in communication. By supplying perceptual analogies for vivid communication, sound-symbolism allows for communication to be *effective*; by providing the lexicon with greater depth and distinction, arbitrariness allows for the *efficient* communication of concepts. The two systems lend themselves better to different communicative uses, which do not preclude each other, and are in fact complementary. The research is slowly leading the field toward a complementary view of language which features both sound-symbolism and arbitrariness, but there are a few obstacles in the way, not least coming up with a widely-accepted and consistently-applied understanding of exactly what sound-symbolism actually is.

### Types of Sound-Symbolism

While arbitrariness is defined by the absolute lack of relation between form and meaning, defining sound-symbolism is somewhat harder; the sheer variety of depth and type of links between form and meaning, both within and across languages, means that there is no simple opposite of arbitrariness. Perniss et al. (2010) and Schmidtke et al. (2014) cover various subtypes of sound-symbolism in detail; a quick overview will be given here. The term *iconicity* is the closest cover-all term for communicative signs showing a resemblance between form and meaning, used as “a blanket term for a broad range of phenomena, including what has been referred to in the literature as sound-symbolism, mimetics, ideophones, and iconicity” (Perniss et al., 2010). Iconicity can be applied to communication in visual, spoken, and other modalities, can be manifested at all levels from phonetics to discourse, and is perhaps even present in animal communication (Hockett, 1959).

In this review paper, we use the term *sound-symbolism* to refer to iconicity in spoken language. Hinton et al. (1994, 2006) define sound-symbolism as “the direct linkage between sound and meaning,” and divide it into *corporeal*, *imitative*, *conventional*, and *synesthetic* sound-symbolism. Cuskley and Kirby (2013) refine the latter two into *conventional* and *sensory* sound-symbolism. Conventional sound-symbolism is the regular correlation between specific sounds or clusters and specific meanings (such as with phonaesthemes). Conventional sound-symbolism can also cover the correlation between sounds and grammatical categories, which is broadly equivalent to what Monaghan et al. (2011, 2014) call *systematicity*. This definition of conventional sound-symbolism has a wider scope than most, as it goes further than Hinton et al. (1994, 2006) who do not consider sound-symbolism as extending to grammatical categories, while Monaghan et al. (2011, 2014) also consider systematicity to be separate from sound-symbolism, which they limit to phonaesthemes and sensory sound-symbolism. Sensory sound-symbolism is a natural connection where the word’s form imitates aspects of the referent within or across modalities, and this imitation is often obvious across languages.

This classification echoes the description of sound systems outlined by Von Humboldt (1836). “Since words always correspond to concepts, it is natural for related concepts to be designated by related sounds.” Von Humboldt lists three ways in which sounds designate concepts: *direct imitation*, which broadly follows imitative sound-symbolism or onomatopoeia; *symbolic designation*, whereby sounds “partly in themselves and partly by comparison with others produce for the ear an impression similar to that of the object upon the soul,” and which most closely resembles sensory sound-symbolism with the acknowledgment of some degree of conventionalism; and *analogical designation*, whereby “words whose meanings lie close to one another are likewise accorded similar sounds; but … there is no regard here to the character inherent in these sounds themselves,” which most closely resembles conventional sound-symbolism driven by statistical association, or systematicity. A closely related distinction is Gasser et al.’s (2010) two-way classification of iconicity as *absolute* or *relative*. *Absolute* iconicity is where there is a direct relation between form and meaning (as in onomatopoeic words for animal sounds). *Relative* iconicity is where related forms are associated with related meanings, as when a contrast between the vowels [i:a] depicts an analogous contrast in magnitude.

Many different terms and definitions have been used for sound-symbolic words, but *ideophone* is now the most widely used and accepted (Voeltz and Kilian-Hatz, 2001). Nuckolls (1999) defines ideophones as “lexicalised sound-imitative words,” while Dingemanse (2012) provides a more specific definition of ideophones as “marked words which depict sensory imagery.” Ideophones typically exhibit sensory sound-symbolism, although there is always some degree of conventionalization involved as well. Thus the Japanese ideophone *kirakira* “glittering” shows sensory sound-symbolism in that reduplication in the word is associated with a continuous meaning and the vowel [i] is associated with brightness, but it also has conventionalized aspects in that not all aspects of its meaning can be deduced from its sounds.

Sound-symbolism is not confined solely to ideophones; in fact, the majority of sound-symbolism research has focused on cross-modal relations between individual sounds and sensory meanings, such as vowels and object size. There are also sound-symbolic links between certain combinations of sounds and meanings. Phonaesthemes are “frequently recurring sound-meaning pairings that are not clearly contrastive morphemes” (Bergen, 2004), such as such as *tw*- in English words like *twist*, *tweak*, *twizzle*, *twirl*, and *twine*. They show a mix of conventional and sensory sound-symbolism (Kwon and Round, 2014), and are thought to be drivers of neologisms in language (Malkiel, 1994). Again, Von Humboldt (1836) wrote of such conventionalized forms having “undoubtedly exerted a great and perhaps exclusive dominance on primitive word-designation … and the new increment is formed by analogy with what is already present.” This philosophical legacy has posed the question of how sound-symbolism constitutes and affects language; it is now the responsibility of modern experimental approaches to bring iconicity out of the wild and into the lab to resolve the argument between Cratylus and Hermogenes with evidence as well as reason.

## Behavioral Experiments

There is a long history of behavioral research on sound-symbolism, most of which has investigated the mappings between consonant/vowel types and the size or shape of visual stimuli in variations on experiments performed by Sapir (1929), Newman (1933), and Köhler (1947). Half a century of Generativism saw sound-symbolism research fall out of favor somewhat, but this approach was brought back into fashion around the turn of the century (Waugh, 1994; Kita, 1997; Ramachandran and Hubbard, 2001; Klamer, 2002), and described in detail in Perniss et al. (2010). To begin with, it was enough simply to show that certain sounds have some kind of effect; this was an important rediscovery which brought sound-symbolism in from the cold and into the wider attention of the field. More recently, there have been several studies in the last few years which have attempted to tease apart the separate roles of vowels and consonants, either by testing participants with individual phonemes or with non-words. These studies have also examined the effect of specific sounds on various different modalities, including strength, light, and taste.

### Forced Choice Tasks With Non-Words

The standard paradigm in behavioral sound-symbolism experiments is the *kiki-bouba* paradigm. Originally developed by Köhler (1947), participants see two shapes—one spiky and one round—and two non-words—*takete* and *maluma* [later adapted to *kiki* and *bouba* by Ramachandran and Hubbard (2001)]. Participants are then asked to say which non-word goes with which shape. Participants generally map the round shape with the “round” non-words (*maluma/bouba*) and the spiky shape with the “spiky” word (*takete/kiki*). Despite the methodologically sparse descriptions in Ramachandran and Hubbard (2001), this effect appears to be strong and consistent, and is the most well-known result showing that the relation between sound and meaning is not entirely arbitrary. This paradigm, and most variations of it, is perhaps the most obvious example of sensory sound-symbolism.

Building on the *kiki-bouba* paradigm, various experiments have found consistent effects with better-controlled stimuli. The paradigm is affected by altering both individual consonants and vowels, but not by mode of presentation, as the effect was consistent regardless of whether the stimuli were presented auditorily or visually (Nielsen and Rendall, 2011). Systematically altering the placement of consonants and vowels in novel words addressed the shortcomings of Ramachandran and Hubbard’s (2001) study, where the 95% success rate was down to the obvious distinction created by the non-words and novel shapes which were deliberately designed to be as different as possible. A follow-up non-word/shape matching experiment revealed a learning bias toward sound-symbolism, albeit a weak one (Nielsen and Rendall, 2012). Two groups of participants were investigated; one which had been implicitly taught a congruent sound-symbolic pattern (plosives and spiky shapes, sonorants and curvy shapes) and one which had been implicitly taught an incongruent sound-symbolic pattern (plosives and curvy shapes, sonorants and spiky shapes). The first group performed above chance in the matching task while the second group performed at chance level, which demonstrates a learning bias toward sound-symbolism. In a novel word generation task (Nielsen and Rendall, 2013), participants were found to use both vowels and consonants to form sound-symbolic associations. Participants used sonorant consonants and rounded vowels for curvy *bouba* figures and plosive consonants and non-rounded vowels for spiky *kiki* figures. Participants also favored vowels with relatively close articulation to the co-articulated consonant (such as a frontal [i] following the strident consonants [t] and [k] and the “frontal” consonants [m], and [n]) and showed a dispreference for combining consonants and vowels which were relatively further apart. This suggested once more that consonants trump vowels when it comes to non-word sound-symbolic perception of visual contours, but that both types of sound do have a role.

The *kiki-bouba* paradigm has also been informative about language in populations different from psychology undergraduate students participating for course credit (Henrich et al., 2010). A first cross-linguistic and cross-cultural replication of Köhler’s (1947) *maluma-takete* paradigm was Davis’s (1961) study of English and Tanzanian children. More recently, Bremner et al. (2013) replicated the *kiki-bouba* paradigm with Himba participants in Namibia for sound-to-shape matching but not taste-to-shape matching. The Himba have no written language and very little exposure to Western culture, which is helpful in ruling out cultural or orthographic effects such as associations with brand names or associations with the shape of the letters (such as how the letter K is spikier than the letter O).

Finally, developmental disorders involving impaired cross-modal integration also affect participants’ accuracy at the *kiki-bouba* paradigm. High functioning autistic participants were significantly worse than non-autistic participants at matching *kiki*-like words to spiky shapes and *bouba*-like words to curvy shapes, although they still categorized the stimuli at above-chance level; low functioning autistic participants performed at chance level (although this may be due to the nature of the task; Occelli et al., 2013). Occelli et al. (2013) theorize that this is linked to a global deficiency in multisensory integration in autistic people, suggesting that the cross-modal correspondence effect is linked to motor and sensory integrative processes in the left inferior frontal gyrus. Dyslexic Dutch speakers, meanwhile, perform above chance at *kiki-bouba* paradigms but worse than non-dyslexic Dutch speakers (Drijvers et al., 2015). This reinforces the claim that cross-modal abstraction is involved in making sound-symbolic links.

### Task Effects

The robustness of the *kiki-bouba* paradigm relies in part on the nature of forced choice. When it uses four target stimuli rather than two, participants are less successful at making congruent sound-symbolic matches (Aveyard, 2012). Moreover, the use of three rounds of testing showed that participants use different strategies depending on whether the paradigm is a two- or four-alternative forced choice task. When there were only two choices, participants used a consonantal sound-symbolic strategy instantly, and general accuracy for incongruent trials improved over three rounds of testing, indicating that participants were able to use separate strategies for congruent and incongruent trials after some experience. When the number of choices was increased to four, participants were less aware of the manipulation and were slower to incorporate consonantal sound-symbolism into their decision making, although this did emerge by the third round. The main effect of linking sonorants to curviness and plosives to spikiness is in line with most behavioral research, but introduces some important variables which show how easily this sensitivity to consonantal sound-symbolism can be affected by experimental set-up.

### Moving Beyond Shape

While the *kiki-bouba* paradigm has been very popular for sound-symbolism research into shape, other experimental approaches are more useful for investigating other sensory modalities. Hirata et al. (2011) found an effect of lightness on sound sensitivity. Participants were better able to identify consonants when they heard and saw congruent sound–light pairings (i.e., voiceless consonants with light visual stimuli, voiced consonants with dark visual stimuli) than incongruent sound–light pairings. However, there was no effect of consonant type when participants had to identify whether a visual stimulus was light or dark.

Links between sound and emotion have also been investigated, but these are more likely to rely on indexical interpretations of affective prosody rather than on iconicity in the sense of structural resemblance (Majid, 2012).

Most of the research presented so far has focused on the properties of consonants, but sensory sound-symbolism with vowels is well-attested too, especially for size (Sapir, 1929). Thompson and Estes (2011) and Thompson (2013) investigated sound-symbolism and object size links by addressing the forced dichotomy of two-alternative forced choice matching in a slightly different way from Aveyard (2012). They showed five different sizes of novel object set against a picture of a cow as a point of comparison, and asked participants to choose the most appropriate name from a selection of three-syllable non-words which varied the number of small-sounding (such as [i]) and large-sounding (such as [a]) vowels. Participants chose non-words with increasing numbers of large phonemes for increasingly large objects, which shows that sound-symbolism marks graded cross-modal mappings rather than just marking contrasts. Meanwhile, it appears that the evidence for an acoustic mechanism for sound-symbolism is stronger than that for a kinaesthetic mechanism, a perennial debate which goes back to Sapir (1929) and Newman (1933). Ohtake and Haryu (2013) performed a series of experiments which separated acoustic features of vowels and the size of the oral cavity while asking participants to categorize the size of a visual object. Participants were faster to categorize object size when hearing the vowels [a] and [i] in congruent conditions, i.e., when [a] was presenting with a large object and [i] with a small object. However, there was no effect when participants categorized object size while holding objects in their mouths to simulate the oral cavity shape made when pronouncing the vowels [a] and [i]. This suggests that the main driver of the effect is the acoustic properties of the vowels, rather than their articulatory properties.

**FIGURE 1:**
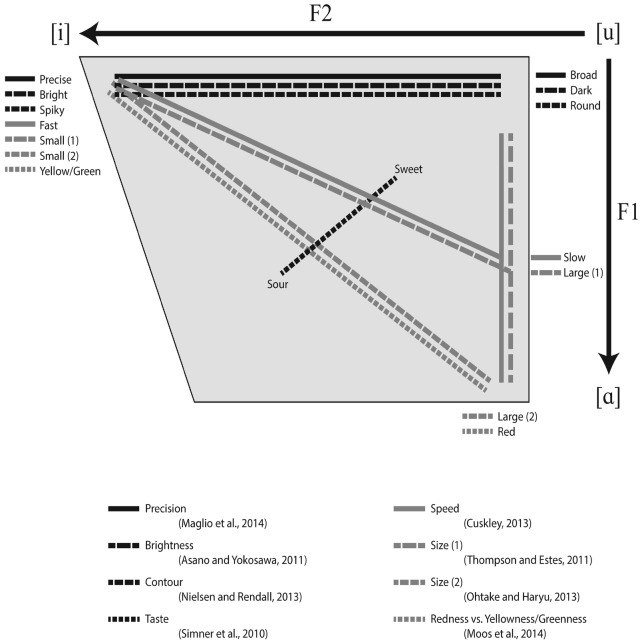
**Diagram of attested cross-modal mappings to linguistic sound represented on typical vowel space**.

The acoustic properties of vowels have also been found to elicit cross-modal correspondences related to taste (Simner et al., 2010). Participants were given taste samples of four taste types—sweet, sour, bitter, and salty—and adjusted four sliders—F1, F2, voice discontinuity, and spectral balance—to create a vowel sound which best fit the taste. Participants consistently assigned lower F1 and F2 frequencies (approximating higher, more back vowels) to sweet flavors and higher F1 and F2 frequencies (approximating lower, more front vowels) to sour flavors, with salty and bitter flavors falling in between. They posit that these patterns may have influenced vocabulary construction for taste terminology. Interestingly, this spectrum does not quite fit along the same lines as most sound-symbolic vowel associations, which tend to run on a spectrum from [i] to [a] as illustrated in Figure 1. Given that Anglophones find it especially hard to describe and discriminate between tastes and smells according to their properties (as opposed to their sources) when compared to other senses (Majid and Burenhult, 2014), perhaps it is to be expected that Anglophone participants may not map sounds onto tastes in the same way as other senses. It is also hard to say what kind of sound-symbolic links drive this effect. It is probably sensory sound-symbolism, but there may be conventional aspects involved; the word *sour* is pronounced with a lower vowel than the word *sweet*, which mirrors the associations made by the participants.

Differences between back vowels and front vowels have been found in various studies. Cuskley (2013) investigated non-words and visual motion by asking participants to direct the motion of a ball to match a non-word. Participants made the ball travel more slowly in response to back vowels, and made the ball travel more quickly in response to front vowels and syllable reduplication with vowel alternation (the apophonic direction of vowel alternation in reduplicated syllables was not tested; forms such as *kigu* and *kugi* were treated as the same). However, whether this mapping is consistent is unclear; Thompson (2013) performed a similar study and found only a small and non-statistical trend toward assigning faster ratings to names containing front vowels.

Maglio et al. (2014) linked front vowels to conceptual precision with two studies on vision and concepts. Participants were asked to perform a geographical analysis of a fictional city. When the city’s name featured more front vowels than back vowels, participants divided the city into smaller, more precise geographic regions, and *vice versa*, which Maglio et al. (2014) refer to as visual precision. Participants were also more precise when asked to describe the actions of a person when there was a front vowel association. They saw a person writing a list and were told that this person was performing a “sheeb task” or a “shoob task”; when asked to describe the person’s behavior, participants replied with conceptual precision about the action in the front vowel condition (e.g., “the person is writing a list” when performing the “sheeb task”), and replied with conceptual breadth about the action in the back vowel condition (e.g., “the person is getting organized” when performing the “shoob task”). This may actually be an indirect measure of the typical vowel-size correspondences, with the participants associating back vowels with size in general and then applying the size distinction to visual or conceptual precision. Maglio et al. (2014) then performed a series of experiments on high versus low-level thought; these linked front vowels to low-level thought and back vowels to high-level thought. Back vowels in an ice-cream product name made people focus on how good it tastes rather than how easily accessible it is; back vowels in a skin lotion product name made people focus on how effective it is, rather than how attractive the packaging is; and back vowels in a back pain treatment made people focus on how long-lasting the pain relief is, rather than how arduous the procedure is. Maglio et al.’s (2014) research provides interesting evidence that specific vowel changes may elicit different mental representations. This probably examines conventional sound-symbolism rather than sensory sound-symbolism, as vowel size does not map onto literal sensory size but a more metaphorical magnitude of abstract concepts.

Some studies have linked cross-modal associations between linguistic stimuli and color to synesthesia. Moos et al. (2014) investigated vowel sound and color associations in synesthetes and control participants. They found that increased F2 (such as in front vowels like /i/) was associated with increased yellowness and greenness on the color spectrum, while increased F1 (such as in open vowels like /ɑ/) was associated with increased redness. This was found in both synesthetes and non-synesthetes, although far more strongly in the synesthetes, which suggests that grapheme-color synesthesia is at least partially based on acoustic properties of the sounds associated to the graphemes, and provides further evidence that synesthesia may be an exaggeration of general cross-modal associations which most people have. Shin and Kim (2014) likewise investigated color associations in synesthetes by comparing the associations of Japanese, Korean, and English graphemes in trilingual synesthetes. Despite the small sample size, they found that color associations were broadly similar across participants and across languages for graphemes which expressed the same sounds, showing that grapheme-color synesthesia for individual graphemes is based on the sounds which the graphemes express. In experiments with synesthetic Japanese speakers, Asano and Yokosawa (2011) found that consonants and vowels independently influence the colors which synesthetes ascribe to the hiragana and katakana Japanese writing systems, and that this effect was not due to visual form. Their results show a tendency for front vowels and voiceless consonants to be associated with brighter colors, and for back vowels and voiced consonants to be associated with darker colors, which follows the general synesthetic patterns set out by Marks (1978). The fact that most of the participants are synesthetic in these three studies makes it hard to say which type of sound-symbolism is under investigation here, but it is likely to be sensory sound-symbolism.

### Summary of Attested Cross-Modal Correspondences

**FIGURE 2:**
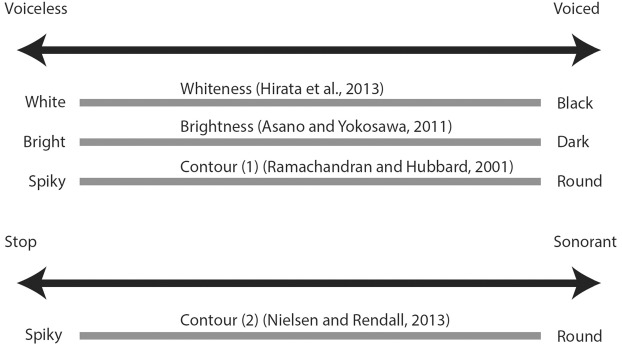
**Diagram of attested cross-modal mappings to linguistic sound for consonant properties**.

Non-word behavioral experiments have been useful in establishing broadly consistent cross-modal associations between sound and other sensory modalities, and these seem to overlap with synesthetic associations. When presenting full non-words, consonants seem to have greater prominence than vowels in terms of what participants perceive and how they formulate sound-symbolic strategies; however, both consonants and vowels do influence participants’ judgments. Voiced consonants and low back vowels are consistently associated with roundness, darkness in color, darkness in light intensity, and slowness (although in the case of voiced consonants, only by comparison with voiceless consonants). Voiceless consonants and high front vowels are consistently associated with spikiness, brightness in color, brightness in light intensity, and quickness. Moreover, vowel height and size is linked with physical size, with low vowels and back vowels being linked to big objects and high vowels and front vowels being linked to small objects. Taste conflates the two acoustic properties of vowels; sweetness is linked with high back vowels and saltiness is linked with low front vowels. This is illustrated in Figures 1 and 2.

### Moving Beyond Non-Words

Despite the progress made with behavioral research on non-words, the insights it provides into language processing are limited. Non-word stimuli are carefully designed to provide maximal distinction between the sensory properties of the referent and the linguistic factors of interest, such as consonant voicing, vowel height and backness, and lip rounding. Not only does this introduce experimenter bias concerning which properties of language are sound-symbolic, it also means that the language stimuli used are not necessarily reflective of spoken language if such maximal distinctions do not occur naturally, and any existing findings may be an overstatement of the cross-modal associations that people make with real language. One way to address this problem is to use existing sound-symbolic words to address the question of how sound-symbolism in natural language is (or is not) associated with other sensory modalities; and among existing sound-symbolic words, ideophones are a prime source of information about sound-symbolic mappings (Dingemanse, 2012).

Most experimental work on ideophones has been conducted using Japanese, which has an extensive, commonly-used and well-documented set of ideophones (Kita, 1997; Hamano, 1998; Akita, 2009a). Most studies have found that participants with no knowledge of Japanese perform significantly above chance at guessing the meaning of ideophones. Oda (2000) performed a series of forced choice tasks with Japanese ideophones on two groups of native English speakers. The first group heard a native Japanese speaker read out the ideophones and were asked to focus on the sound before performing the tasks. The second group heard a native Japanese speaker read out the ideophones and were then asked to pronounce the words themselves before performing the tasks. The two tasks were picking the correct ideophone out of three options for one English definition, and matching two minimal pair ideophones to the two English definitions, which were accompanied by illustrations of the texture or movement. Both groups could guess the meaning of the ideophones at an above chance level of accuracy, and this accuracy was modulated by articulation; the group which pronounced the words themselves were significantly better at matching unfamiliar ideophones to English definitions. In opposition to studies such as Ohtake and Haryu (2013), Oda’s (2000) result suggests that articulation does play a role in establishing the form-meaning relationship of ideophones. The question over whether sound-symbolism is driven by acoustic *or* articulatory mappings is perhaps too reductive; it seems that both mechanisms are involved depending on the nature of the task.

Iwasaki et al. (2007a) conducted similar experiments with Japanese pain vocabulary, and found that non-Japanese speakers could accurately categorize ideophones expressing pain according to the type of pain they express. However, Japanese sound-symbolism is not always entirely transparent to other speakers. In another study, Iwasaki et al. (2007b) found that English speakers with no knowledge of Japanese could make accurate semantic judgments about ideophones which referred to specific sound qualities but the same speakers made very different semantic judgments about ideophones concerning beauty and pleasantness. It is unclear whether this is due to the fact that sound-to-sound mappings do not cross modalities and are therefore more transparent, whether these particular ideophones expressing beauty were just more on the conventional side of the continuum and therefore less obviously iconic, or due to cultural differences over what constitutes beauty.

Iwasaki et al. (2007b) further found that English speakers were relatively better at categorizing ideophones describing manners of laughter (e.g., giggling and chuckling according to semantic dimensions like pitch and gracefulness) than ideophones describing manners of walking (e.g., strolling and lumbering according to semantic dimensions like pace and steadiness). Iwasaki et al. (2007b) attributed this to the same kind of vowel and consonant voicing contrasts which have been found in non-word studies, such as large vowels being linked with large strides and loud laughs. However, it also shows that ideophones are not completely intuitive to speakers of other languages and depend in some part on the specific semantic context provided by the experimental set-up. In a developmental study, Imai et al. (2008) generated some novel ideophones for manners of motion based on Hamano’s (1998) phonosemantic classification of Japanese ideophones, and Japanese adult participants completely agreed with the novel ideophones’ intended meanings. This supports the idea that at least some of the sound-symbolic patterns in Japanese ideophones are sufficiently systematic enough to be productive (Oda, 2000; Yoshida, 2012). When naïve English speakers were tested with these novel ideophones, the intended meanings were still categorized at above chance level, thus confirming previous behavioral research on Japanese ideophones with novel forms. All of these studies with Japanese ideophones show that there is enough sensory sound-symbolism in ideophones for speakers of other languages to be sensitive to the meanings, and that there may be additional conventional sound-symbolism in ideophones which is more informative for native speakers.

### The Role of Prosody

Similar above chance categorization patterns have been found with ideophones in various languages, not just Japanese. Mitterer et al. (2012) took ideophones from five languages across five semantic domains, and presented naïve participants with four versions of the stimuli in two-alternative forced choice tasks—the original ideophone recordings, a rich resynthesis using the original recordings’ phoneme durations and prosody, a phoneme-only resynthesis and a prosody-only resynthesis. Ideophones in the original recordings and in the rich resynthesis condition were both categorized at above-chance accuracy, but ideophones in the phoneme-only and prosody-only resynthesis conditions were not. This indicates that both phonemes and prosody are important for cross-linguistic effects of iconicity. This finding is corroborated by evidence that around 80% of ideophones are given special prosodic attention and emphasis in natural speech—prosodically foregrounded (Dingemanse, 2013)—and that certain prosodic profiles in non-words can have reliable semantic associations (Nygaard et al., 2009b).

Some non-ideophonic lexical words also show these effects. Kunihira (1971) conducted experiments using apparently arbitrary Japanese words in forced choice tests and found that English speakers were able to accurately categorize them, even though they were not ideophones. Responses were most accurate when the words were pronounced with “expressive voice,” i.e., exaggerated prosody. This suggests sound-symbolic interpretations can be elicited even for arbitrary words—a viewpoint that reinforces the crucial role of expressive prosody. Nygaard et al. (2009a) used Kunihira’s stimuli in a learning task, and found that English speakers were quicker to learn and quicker to respond to Japanese words paired with correct English translations (e.g., *hayai* and *fast*) than when paired with opposite (e.g., *slow*) or unrelated (e.g., *blunt*) English translations. Nygaard et al. (2009a) stop short of linking particular sounds or properties of the words to particular meanings, instead suggesting that reliable sound-meaning mappings—regardless of whether this sound-symbolism is sensory (i.e., presumably recognizable across languages) or conventionalized (i.e., recognizable only within a particular language)—“may constrain novel word learning and subsequent word retrieval and recognition by guiding processing to properties and meaning within a particular semantic context.”

The same research group expanded the scope of this research to include antonym contrasts in 10 different languages; monolingual English speakers allocated the antonyms correctly at above chance level in two-alternative forced choice testing, although consistency varied across individual items and may indicate the inherent probabilistic variability in the degree of sound-symbolism in supposedly arbitrary words (Namy et al., submitted; Tzeng et al., submitted). These findings were partially replicated in a study comparing synesthetes and non-synesthetes, which found that both groups guessed certain meanings at above chance accuracy, and that the synesthetes did so more strongly than the non-synesthetes (Bankieris and Simner, 2015). However, there are two crucial caveats with these stimuli. Firstly, six of the 10 languages used in these studies are rich in ideophones and poor in ordinary adjectives (Indonesian, Korean, Tamil, Mandarin, Turkish, and Yoruba), which means that this study may well have indirectly studied ideophones rather than arbitrary antonyms. Secondly, the four non-ideophonic languages (Dutch, Albanian, Gujurati, and Romanian) are all Indo-European; this means that they cannot be treated as independent because of potentially shared linguistic features, and moreover their meanings may be more transparent to native English speakers if they are cognates, especially in the case of Dutch and Romanian. Unfortunately, these studies are not yet publicly available (despite their crucial role in other published work), and so we cannot do more than speculate here.

## Developmental Experiments

While the extensive behavioral literature attests that sound-symbolism has persistent and varied effects on language processing and use, a frequent criticism is that these patterns of association are conditioned because of orthographic influences; people might only consider the sound [b] to be rounder than the sound [k] because the letter *b* is rounder than the letter *k*. However, studies on early language development have shown that this is not the case. Studies with pre-literate children and young infants rule out such orthographic effects. Developmental experiments with infants also provide a different window into sound-symbolism from learning experiments with adults. Experiments with infants examine existing cross-modal associations and how infants exploit these during early language development, whereas learning experiments with adults examine how sound-symbolism affects memory, and are necessarily influenced by the adults’ first language.

### Mixed Results for kiki-bouba Paradigms

The *kiki-bouba* paradigm, with its sensory sound-symbolism links, can be easily adapted for infants and young children, although results have been mixed. Ozturk et al. (2013) and Fort et al. (2013) tested 4-month-old infants with preferential looking procedures, using fully reduplicated non-words with no word-internal vowel contrasts (e.g., *kiki*, *bubu*). Ozturk et al. (2013) presented one shape together with one auditory non-word and measured gaze duration, while Fort et al. (2013) presented two shapes side by side together with one auditory non-word and investigated whether infants preferred looking at a particular shape. The additional complexities of Fort et al.’s (2013) experimental set-up proved to be too much for the infants, as they found no preferential looking effects; they “tentatively argue that the complexity of their design might have masked the infants’ emerging sound-symbolic matching abilities.” However, Ozturk et al. (2013) found that infants looked for longer durations at shapes which were presented with incongruent non-words. Moreover, they found that this only happened for non-words where both vowels and consonants were typically sound-symbolic; the infants would match *bubu* with the curvy shape and *kiki* with the spiky shape, but would not make the same distinctions when comparing *kiki* and *kuku* or *bibi* and *bubu*. The adult control group, on the other hand, only needed either a vowel contrast or a consonant contrast to make cross-modal associations. When taken together, these results suggest that there is an effect of sound-symbolism in infants, but that it needs both consonants and vowels to make the stimuli maximally distinct and that only very straightforward designs may detect the effect. This also appears to show that infants are less sensitive to sound-symbolic contrasts than adults are, which implies that increased exposure to language in fact increases sensitivity to sound-symbolic associations. This is supported by a study on pitch-size associations in 4- and 6-month-old infants, which found that 6-month-old infants make typical associations between pitch and size while 4-month-old infants do not (Fernández-Prieto et al., 2015). The apparent conflict in results between Fort et al. (2013) and Ozturk et al. (2013) shows that iconicity may be strong enough for infants to detect, but not strong enough for this effect to persist through more complicated tasks.

Maurer et al. (2006) replicated Ramachandran and Hubbard’s (2001) *kiki-bouba* results with 2.5-year-old children, which ruled out orthography as a confound as these children could not yet read. Spector and Maurer (2013) developed this experiment with slightly updated stimuli, using fully reduplicated non-words with no word-internal vowel contrasts rather than the typical *kiki-bouba* words used in the previous study. The toddlers were presented with two visual shapes, and then asked by an adult to point to the non-word of interest (e.g., “can you point to the *koko*?”). As predicted, the toddlers associated curvy shapes with rounded vowels and spiky shapes with non-rounded vowels. One possible factor is the direct interaction with an adult experimenter rather than pre-recorded stimuli. Nygaard et al. (2009b) have established that adults use exaggerated and semantically-predictable prosodic profiles when pronouncing non-words in child-directed speech, and this may have provided the kind of prosodic foregrounding which helps to identify ideophones in natural language.

There have also been several developmental studies on the acquisition and use of Japanese ideophones, which show that both Japanese and non-Japanese children are highly sensitive to the sound-symbolic properties of Japanese ideophones. Iwasaki et al. (2007b) cite Ishiguro (1993), who found that children create their own idiosyncratic ideophones before fully acquiring conventional ones, and that children acquire ideophones expressing sound before acquiring ideophones expressing motion, shape, psychological states, or other sensory modalities. This ties in with Iwasaki et al.’s (2007b) and Oda’s (2000) research, which showed that participants with no knowledge of Japanese were more accurate at categorizing ideophones expressing sound, and confirms the prevalence of sensory sound-symbolism in ideophones.

### The Sound-Symbolic Bootstrapping Hypothesis

Imai et al. (2008) created novel Japanese ideophonic motion verbs and tested them on Japanese and English-speaking adults (as described in the behavioral section). They then tested 25-month-old Japanese children with a verb learning task, and found that the children could generalize the ideophonic verbs to new situations, but could not do the same for the non-sound-symbolic verbs. Imai et al. (2008) concluded that sound-symbolism provides a scaffold on which children can map semantic and syntactic information. Echoing Gentner and Boroditsky’s (2001) arguments that actions unfold over time and are impermanent whereas objects are stable, which is why children tend to focus on objects and tend to acquire nouns first, Imai et al. (2008) propose that the sound-symbolic scaffolding provided by the ideophonic verbs helps children to isolate the action and therefore facilitates verb learning. Kantartzis et al. (2011) replicated Imai et al.’s (2008) results in experiments with English children using the same novel verbs based on Japanese sound-symbolic patterns. This provided evidence toward a cross-linguistic—or, perhaps more accurately, language-independent—early sensitivity toward sound-symbolism, and also shows that Japanese ideophones contain sensory sound-symbolism and not just conventional sound-symbolism. Kantartzis et al. (2011) also point out that it is unclear what exactly the English children recognize as sound-symbolic; it could be the phonetics, the phonotactics, the prosody, or a combination of all three.

Yoshida (2012) developed the paradigm further and carried out more extensive tests, making several important points. Firstly, sound-symbolism aided verb acquisition in Japanese and English children equally, despite the Japanese children’s greater exposure to and familiarity with the Japanese mimetic-style novel verbs. Secondly, this equal language-independent sensitivity to sound-symbolism exists despite the vast difference in general iconic input between Japanese (where parents make extensive use of ideophones to children) and English (where parents do use a lot of onomatopoeia to children, but they do so more idiosyncratically and less often than Japanese parents do). Thirdly, by including both novel verbs and novel actors in the task, she showed that the sound-symbolic scaffolding proposed by Imai et al. (2008) Imai and Kita (2014) helps children to isolate the action by excluding the identity of the actor, rather than just by focusing on the action. Yoshida (2012) proposes that infants are universally sensitive toward sound-symbolism, but this sensitivity attenuates in adulthood as their native language’s conventionalized forms dictate which possible forms of sound-symbolism are acceptable; this mirrors the well-established pattern of infant sensitivity to cross-linguistic phonemic differences, which attenuates with age. The sound-symbolic bootstrapping hypothesis is also supported by ideophone usage studies, which have shown that Japanese children as young as 2 years old use ideophonic verbs frequently and productively (Akita, 2009b) and that Japanese parents are five times more likely to use ideophones to children than they were to other adults when describing the same scene (Maguire et al., 2010). The finding that ideophones are more geared toward children initially appears to sit uncomfortably with the finding of Ozturk et al. (2013), which suggested that infants were less sensitive to sound-symbolism than adults. However, perhaps a reasonable middle ground is that children are more sensitive to sound-symbolism as long as there are enough sources in the input to make associations from, while adults are less sensitive to sound-symbolism in terms of forming associations but can form associations from a more limited input.

Finally, Laing’s (2014) reanalysis of a longitudinal case study (Elsen, 1991) provides another example of how sound-symbolism bootstraps language acquisition. Laing examined Elsen’s detailed dataset of German infant Annalena and investigated the development and role of onomatopoeic forms. Annalena used onomatopoeic forms extensively, constituting almost 40% of her vocabulary at 11 months, but the relative proportion of onomatopoeia in Annalena’s vocabulary tailed off with age. Annalena systematically replaced onomatopoeic forms with conventional words according to her phonological ability, meaning that onomatopoeic forms were retained longer when their conventional forms were phonologically more difficult. This shows how both sensory and conventional sound-symbolism in infancy works alongside the developing lexicon and can bootstrap phonological development.

## Neuroimaging Experiments

Behavioral research into sound-symbolism has been instrumental in telling us that there is a robust effect of sound-symbolism on language tasks, and that this effect can be modulated by various different linguistic changes. However, neuroimaging research is needed to establish how the brain recognizes, processes, and constructs sound-symbolism. There has been far less neuroimaging research on sound-symbolism than behavioral, but the handful of existing studies make interesting suggestions about sensory embodiment, synesthesia, and multisensory integration.

### ERP and fMRI Evidence

Some neuroimaging experiments on ideophones have essentially used behavioral paradigms with simultaneous EEG recording to investigate ERPs. Kovic et al. (2010) conducted a novel word learning experiment, which established that participants were quicker to identify novel objects with congruent sound-symbolic non-word names than incongruent or arbitrary non-word names. They then tested two groups of participants; one group learned congruent sound-symbolic names for pointy and round objects (i.e., *shick* for a pointy object and *dom* for a round object), the other group learned incongruent sound-symbolic names (i.e., *shick* for a round object and *dom* for a pointy object). The experiment presented a name auditorily and then an object visually, and the participants had to decide whether the object and name matched. The first group were quicker to identify correct conditions and quicker to reject incorrect conditions than the second group, which corroborates other behavioral evidence that sensory sound-symbolic congruence has an object recognition facilitation effect. Moreover, objects with congruent sound-symbolic names elicited a stronger negative wave than incongruent ones in the 140–180 ms window after the presentation of the object. This effect was observed at the occipital regions, home of the visual cortex, and Kovic et al. (2010) suggest that the early negativity represents auditory-visual integration during early sensory processing.

Arata et al. (2010) used the *kiki-bouba* paradigm on 12-month-old infants, simultaneously presenting a shape and a non-word in congruent and incongruent conditions. The infants were found to be sensitive to sound-symbolic matches and mismatches, showing differentiated wave patterns across both conditions after 200 ms post-stimulus. This may have been the P2, an ERP component which has been linked to phonological and semantic analysis. Arata et al. (2010) claim that their results support the claim that infants are synesthetic or like synesthetes (Maurer and Mondloch, 2004), potentially due to having more cortical connections than adults do, resulting in their ability to detect sound-symbolism. Asano et al. (2015) performed a similar experiment on 11-month-old infants, this time presenting the stimuli sequentially; the infants were first shown a spiky or curvy novel object, and then heard the non-word *kipi* or *moma*. This study found a later effect, with more negative ERPs in the 400–550 ms window for incongruent stimuli compared to congruent stimuli. Asano et al. (2015) argue that infants use sensory sound-symbolic congruency to anchor novel sounds onto meaning, thus enabling them to establish that linguistic sounds have real world referents.

There are fewer neuroimaging experiments specifically aimed at revealing the brain locations associated with ideophone use and understanding, probably because of the relative lack of knowledge of ideophones outside the field of linguistics. However, a few neuroimaging studies using ideophones do exist. Osaka and his group conducted a series of fMRI studies (Osaka et al., 2003, 2004; Osaka and Osaka, 2005, 2009; Osaka, 2009, 2011), which show that Japanese ideophones activate the relevant sensory cortical areas. Ideophones expressing laughter activate the “laughter module” (Osaka et al., 2003) across the visual cortex, extrastriate cortex, and the premotor cortex, and also the striatal reward area. Ideophones expressing pain (e.g., *chikuchiku* for a needle-prick kind of pain, *gangan* for a throbbing headache) activate the cingulate cortex, the part of the brain which also processes actual pain. Ideophones expressing crying (e.g., oioi for *wailing*, *mesomeso* for sniveling) activate similar areas to the laughter ideophones, suggesting that crying and laughing are processed as positive and negative equivalents, but they also activate the inferior frontal gyrus and anterior cingulate cortex in the same way as the pain ideophones, suggesting that implied crying “involves some degree of concomitant emotional pain” (Osaka, 2011). Ideophones suggestive of gaze direction and manner of walking activate the frontal eye field and extrastriate visual cortex respectively. All of these ideophones activate the visual cortex and premotor cortex, which Osaka’s group argue is responsible for the vividness of the mental imagery conjured up by ideophones. However, the main limitation with these studies is that they all compared ideophones to non-words. As arbitrary words will also activate relevant sensory areas of the cortex when compared with non-words (Zwaan, 2004), this is uninformative about the special properties of sound-symbolism.

### Ideophones Versus Arbitrary Words in Natural Language

Two neuroimaging studies have directly compared ideophones and arbitrary words. Lockwood and Tuomainen (2015) used EEG to investigate the difference between ideophonic adverbs and arbitrary adverbs by presenting Japanese speakers with sentences where the only difference was whether the adverb was sound-symbolic or not. Participants performed an unrelated sentence judgment task and were unaware of the nature of the experiment. The ideophones elicited a greater P2 and a late positive complex, both of which are in line with Arata et al.’s (2010) and Asano et al.’s (2013, 2015) findings. Lockwood and Tuomainen (2015) argue that the greater P2 in response to the ideophones represents the multisensory integration of sound and sensory processing. They also claim that while this effect is due to cross-modal associations rather than representative of true synesthesia, the same neural mechanisms may be involved. They speculate that it is the distinctive phonological profile of ideophones which enables, or engages, the multisensory integration process. This is also in line with the conclusions of Occelli et al.’s (2013) behavioral study on autistic participants.

Kanero et al. (2014) performed two fMRI studies where participants watched animations while simultaneously hearing ideophones or arbitrary words with related to a particular modality—motion in the first experiment and shape in the second. They observed that words which participants rated as closely matching the animations elicited greater activation across the cortex than low-match words. The right posterior superior temporal sulcus (rpSTS) was activated specifically in response to ideophone trials, and not arbitrary word trials. Kanero et al. (2014) take this to mean that the right posterior STS is a critical hub for processing Japanese ideophones, and possibly sound-symbolism in general. They argue that this goes beyond simple embodiment, as the rpSTS is not a perceptual or sensorimotor area related to the word meaning. Instead, Kanero et al. (2014) suggest that ideophones have a dual nature; part arbitrary linguistic symbol, part iconic symbol, and that the posterior STS works as a hub of multimodal integration. This is in line with a long tradition in the ideophone literature that emphasizes the combination of iconic aspects (such as vowel size contrasts) and arbitrary aspects (such as conventional word forms) in ideophones (e.g., Diffloth, 1994). However, as ideophones contain both sensory and conventional sound-symbolism, it is difficult to tease apart the separate contributions of each type with native speakers.

There has also been a study which used fMRI and fractional anisotropy (FA) to investigate sound-symbolism in apparently arbitrary words. Using the same antonym stimuli and experimental set-up as Namy et al., (submitted) and Tzeng et al., (submitted), Revill et al. (2014) found that there was increased activation in the left superior parietal cortex in response to words which participants found sound-symbolic compared to words which they did not. Furthermore, they found a correlation between functional anisotropy in the left superior longitudinal fasciculus and participants’ individual sensitivity to sound-symbolism. Revill et al. (2014) argue that sound-symbolic words engage cross-modal sensory integration to a greater extent than arbitrary words, and that this cross-modal sensory integration is what facilitates word to meaning mappings (although due to the caveats mentioned above, it is not quite clear what kind of sound-symbolism is under investigation here). They also argue that these correspondences may reflect some form of iconicity or embodiment, but do not speculate whether the main driver of the sound-symbolic effect is acoustic or articulatory.

Finally, Meteyard et al. (2015) investigated the phonological and semantic basis of iconicity with aphasic patients, and used it to addressed theoretical questions rather than just demonstrating an effect. They tested left-hemisphere aphasic patients with four aphasia assessment tests which assess phonology, semantics, and the combination of phonology and semantics, and looked at the processing differences between iconic and non-iconic English words (which are mostly conventionally sound-symbolic with some sensory sound-symbolic properties). Aphasics had an especially consistent processing advantage for iconic words in auditory lexical decision and reading aloud tasks, which specifically involve the mapping between phonology and semantics rather than either phonology or semantics alone. They present two potential theoretical implications, which are not mutually exclusive. Firstly, iconic words may have additional connections from the semantic system to modality-specific features, meaning that iconic words are more robust in aphasic patients because they are represented with greater redundancy within the language system itself. This means that the iconic word processing advantage is protected from damage in a similar way to high frequency, high imageability, and early acquired words. Alternatively, iconic words may be represented by direct connections between phonological form and modality-specific information. This is in line with both the embodiment semantics literature, which claims that iconic words have an extra route to activate experience, and the neuroimaging work of Kanero et al. (2014); under this account, the iconic word processing advantage in aphasics is because iconic words are additionally processed in cross-modal integration brain areas, including right hemisphere regions which are unaffected by left hemisphere damage. This study is probably the best account of how iconicity mediates between semantics and phonology rather than being specific to one or both.

## Summary and Future Directions

The wealth of research on sound-symbolism in the last few years has consolidated three main findings. Firstly, people consistently make multiple cross-modal sensory associations to specific sounds under experimental conditions, and the direction of the cross-modal sensory association—light or dark, fast or slow, etc.,—is related to vowel height, vowel size, and consonant voicing of the sounds involved. Secondly, people can consistently guess the meanings of sound-symbolic words in foreign languages at an above chance level, and that this is related to phonemes and prosody. Thirdly, children are sensitive to sound-symbolism and that ideophones help children acquire verbs (or at least, verbal meanings in the domain of motion) regardless of which language they are learning, meaning that children’s sensitivity to ideophones is likely to be driven by the sound-symbolic phonemes and prosody. There are not yet enough neuroimaging experiments on sound-symbolism to make solid conclusions, but so far it appears that sound-symbolic words activate sensory areas more strongly than arbitrary words and that the processing of sound-symbolic words appear to involve some kind of multisensory integration (or at least more multisensory integration when compared to arbitrary words).

### From Observation to Explanation

The vast majority of these studies have focused on showing that there *is* an effect and have strongly made the case for sound-symbolism; the next step is to investigate how this effect *works*. Prior work has supplied several important pieces of the puzzle. There are linguistic typologies and frameworks for understanding sound-symbolism, such as those of Hinton et al. (2006), Perniss and Vigliocco (2014), Dingemanse (2012), and Cuskley and Kirby (2013). There are some cognitive accounts of structure mapping (Gentner, 1983), of the mental faculties for sound-symbolism (Marks, 1978; Ramachandran and Hubbard, 2001), and of how sound-symbolism scaffolds language acquisition (Imai and Kita, 2014). There is also a host of psychological evidence from cross-modal correspondences. However, two crucial missing pieces in the literature are specific hypotheses of how neural mechanisms may support sound-symbolism, and solid neuroimaging evidence which tests them.

Broadly speaking, psychological studies have addressed the question of which particular sounds have which particular cross-modal correspondences, while linguistic studies have addressed the question of what properties sound symbolic words have which make them sound-symbolic. The current challenge in sound-symbolism research is to pull together the different strands of research into one coherent field. Linguistic, psychological, and cognitive research programs have individually made predictions about the form, use, and function of sound-symbolism; this is now a perfect opportunity for cross-disciplinary collaboration to develop a neuroscientific model of sound-symbolism which makes predictions that can be empirically tested with neuroimaging methods.

### Interdisciplinary Integration

One attempt at interdisciplinary integration is when Ramachandran and Hubbard (2001) used the *kiki-bouba* paradigm to inform their more general synesthetic bootstrapping model of language evolution. They postulate that there is a synesthetic correspondence between visual object shape represented in the inferior temporal lobe and sound represented in the auditory cortex, and that this synesthetic correspondence may either happen through direct cross-activation or may be mediated by the angular gyrus. The first possibility has been interpreted as predicting that relevant sensory areas would be more strongly activated for sound-symbolic words compared to arbitrary words; the second possibility predicts that the angular gyrus would be more strongly activated for sound-symbolic words compared to arbitrary words. Both of these hypotheses can be built on with further neuroimaging work, but of the sound-symbolism experiments that do mention it, they tend either show that there is a significant effect and move on, or they hedge their conclusions by suggesting that there may be a synesthetic or embodiment mechanism without elaborating on how it might work.

Perniss and Vigliocco (2014) also provide a relatively fleshed out model. They propose that iconicity exists to provide the link between linguistic form and human experience by establishing reference and displacement through sensorimotor embodiment of linguistic form, and that the cross-linguistic variability in iconicity shows how different languages strike a balance between two basic constraints—the need to link language to human experience and the need for an efficient communication system. This suggestion provides fertile ground for hypothesis testing, especially with language development literature which can be framed in terms of investigating the emergence of reference and displacement with respect to iconicity. The next step for this model is to hypothesize how the brain processes sound-symbolism and cross-modal correspondences. Perhaps there is a role here for Meteyard et al.’s (2015) suggestion that iconic words may be supported by additional connectivity between semantic or phonological representations and perceptuo-motor information.

Recent research on sound-symbolism has established that sound-symbolism is widespread across languages, that it has cross-modal correspondences with other senses, that this has an effect on behavior and development, and that it elicits distinct brain signals. We are now at an exciting juncture where we can start approaching this phenomenon from an integrated interdisciplinary perspective. Ideophones and sound-symbolism from natural languages provide us with opportunities for the experimental investigation of the role of sound-symbolism in meaning, interpretation, and perception. Through hypothesis-testing and model-building, these experiments will help contribute to a cumulative science of sound-symbolism in human language.

### Conflict of Interest Statement

The authors declare that the research was conducted in the absence of any commercial or financial relationships that could be construed as a potential conflict of interest.

## Footnotes

1 However, this entire debate was not conducted out of academic curiosity; rather, Cratylus had told Hermogenes that Hermogenes was not his real, natural name, assigned as it was by his parents. Thus provoked, Hermogenes became the first documented proponent of arbitrariness, arguing that any given group of people can determine their own labels for concepts and if he calls himself Hermogenes then he has the absolute right to do so since it is only a label for the person he is, thank you very much.

2 Socrates concludes the debate by saying that it is far better to study the things themselves rather than their names, a suggestion which is somewhat less useful for models of language.
