# Corrigendum: Iconicity in the lab: a review of behavioral, developmental, and neuroimaging research into sound-symbolism

**DOI:** 10.3389/fpsyg.2015.01624

**Published:** 2015-10-19

**Authors:** Gwilym Lockwood, Mark Dingemanse

**Affiliations:** ^1^Neurobiology of Language Department, Max Planck Institute for PsycholinguisticsNijmegen, Netherlands; ^2^Language and Cognition Department, Max Planck Institute for PsycholinguisticsNijmegen, Netherlands

**Keywords:** iconicity, sound-symbolism, neuroimaging, psycholinguistics, linguistics, ideophones, synesthesia, cross-modal correspondence

In the original article, the following sentence:

“They found that increased F2 (i.e., higher vowels) was associated with increased redness on the color spectrum, while increased F1 (i.e., lower vowels) was associated with increased yellowness.”

is incorrect. It should be:

“They found that increased F2 (such as in front vowels like /i/) was associated with increased yellowness and greenness on the color spectrum, while increased F1 (such as in open vowels like /ɑ/) was associated with increased redness.”

This means that Figure 1 is also incorrect. A new Figure 1 is provided here.

The original article was updated.

**Figure 1:**
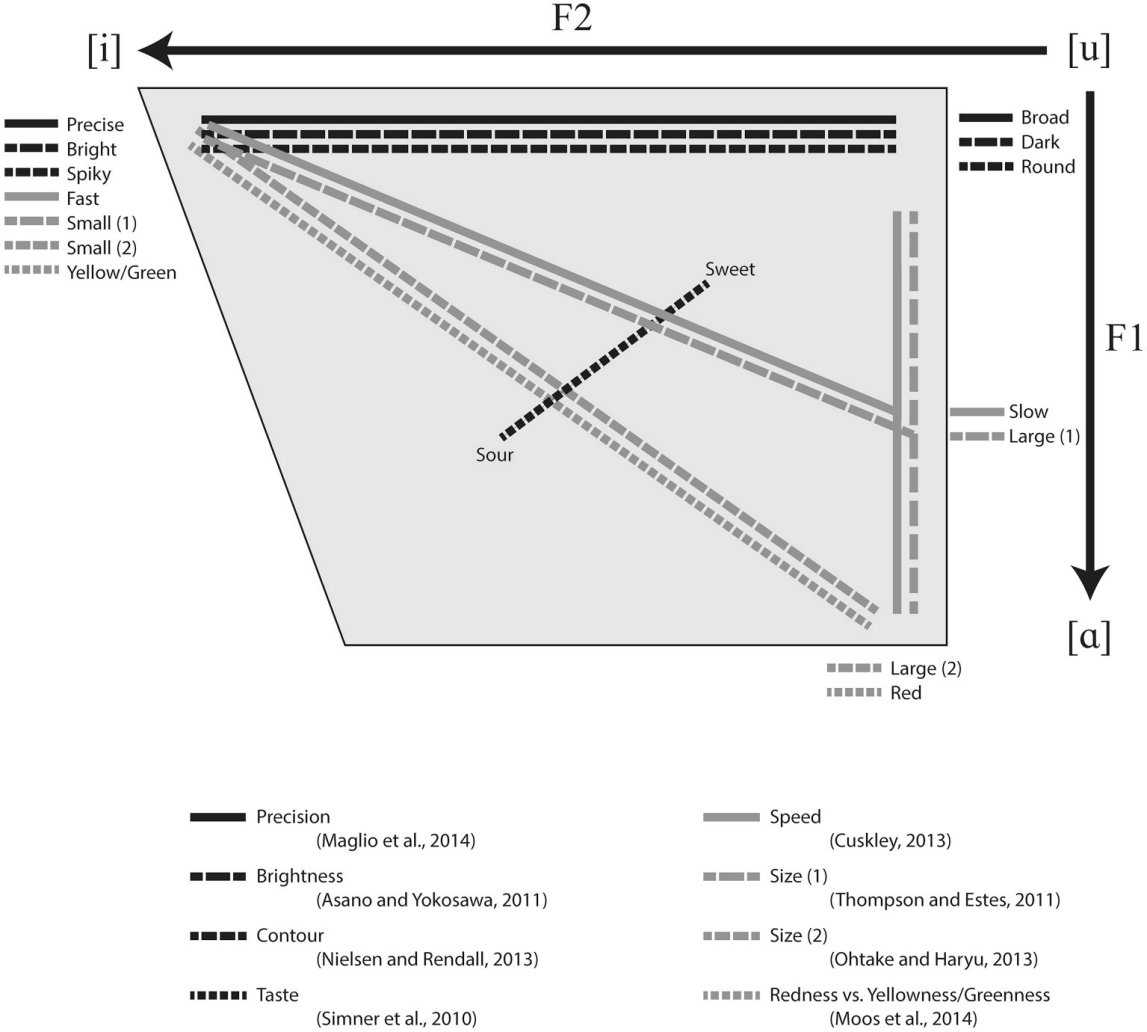
**Diagram of attested cross-modal mappings to linguistic sound represented on typical vowel space**.

## Conflict of interest statement

The authors declare that the research was conducted in the absence of any commercial or financial relationships that could be construed as a potential conflict of interest.

